# Ferroptosis and its current progress in gastric cancer

**DOI:** 10.3389/fcell.2024.1289335

**Published:** 2024-02-28

**Authors:** Zhenqi Yue, Yiwu Yuan, Qi Zhou, Jie Sheng, Lin Xin

**Affiliations:** ^1^ Department of General Surgery, The Second Affiliated Hospital of Nanchang University, Nanchang, Jiangxi, China; ^2^ Jiangxi Province Key Laboratory of Molecular Medicine, The Second Affiliated Hospital of Nanchang University, Nanchang, Jiangxi, China

**Keywords:** ferroptosis, gastric cancer, reactive oxygen species, mechanism, research status

## Abstract

Gastric Cancer (GC) is a prevalent malignancy within the digestive tract, ranking as the fifth most common malignant tumor worldwide. It is characterized by clinical features such as a tendency for metastasis and an unfavorable prognosis. Ferroptosis, a recently identified form of cell death, represents a novel mode of cellular demise that diverges from the traditional concepts of necrosis and apoptosis. Numerous studies have found that ferroptosis plays a significant role in the proliferation, metastasis, drug resistance, and microenvironment regulation within GC. This review summarizes the mechanism of ferroptosis and its role in the occurrence and development of GC cells. It provides examples demonstrating how various anti-tumor drugs can induce ferroptosis in GC cells. Additionally, it summarizes the potential application value of ferroptosis in the future treatment of GC.

## 1 Introduction

GC, as one of the top five malignancies globally, ranks fifth in terms of its global incidence and third in cancer-related mortality (Wang et al., 2022b). Presently, the treatment of GC primarily involves surgery, complemented by adjuvant chemotherapy, targeted therapies, and other measures. However, for advanced-stage GC, especially in late-stage patients, the treatment outcomes remain less than satisfactory (Ogawa et al., 2021; Sugezawa et al., 2022). Simultaneously, investigations indicate a gradual increase in the incidence of GC among young individuals (Petryszyn et al., 2020). Presently, the mechanisms underlying the occurrence and progression of GC remain incompletely understood. Delving deeper into exploring the pathogenesis of GC holds a guiding significance for the treatment of later-stage GC. Ferroptosis, an iron-dependent form of programmed cell death characterized by lipid peroxidation accumulation, intricately regulates the biological processes of malignancies (Lu et al., 2022). Various studies have demonstrated that ferroptosis plays a crucial role in GC’s occurrence, progression, treatment, and prognosis. Therefore, we provide a concise overview of the mechanisms underlying ferroptosis and its involvement in the occurrence, development, drug resistance, and therapeutic aspects of GC. Additionally, we summarize and prospect the potential application value of ferroptosis in the treatment of GC.

## 2 The definition and characteristics of ferroptosis

Ferroptosis, a novel form of iron-dependent form of programmed cell death, is triggered by iron overload, leading to the accumulation of reactive oxygen species (ROS) and lipid peroxidation (Du and Guo, 2022). Compared to other types of cell death such as necrosis, apoptosis, and autophagy, ferroptosis exhibits distinct morphological characteristics, mechanisms of occurrence, and genetic features. Morphologically, cells undergoing ferroptosis exhibit reduced cell volume, intact cell membranes without blebbing, denser mitochondrial membranes compared to normal mitochondria, decreased or vanished mitochondrial cristae, rupture of the mitochondrial outer membrane, with no apparent morphological changes in other organelles (Xie et al., 2016; Doll and Conrad, 2017; Sugezawa et al., 2022). The cell nucleus in ferroptotic cells remains intact, setting it apart from apoptosis (Rishi et al., 2021). Ferroptosis plays a widespread role in regulating various pathophysiological processes in the human body, including tumor cell death, neurodegenerative diseases, tissue ischemia/reperfusion injury, T-cell immunity, and more (Xie et al., 2016; Yu et al., 2023). Within ferroptotic cells, the excessive accumulation of reactive oxygen species (ROS) and lipid peroxides results in the oxidative destruction of phospholipid molecules containing polyunsaturated fatty acids (PUFAs) in cell membranes and organelle membranes. This, in turn, leads to membrane damage (Hassannia et al., 2019; Yu et al., 2023). Research by Riegman et al. confirms that the cellular iron death signal initially spreads in a wave-like manner within a cell population, subsequently leading to cell swelling downstream in the signaling cascade, ultimately culminating in cell rupture mediated by the plasma membrane pores (Riegman et al., 2020; Rishi et al., 2021). The induction of intracellular iron death predominantly occurs at the endoplasmic reticulum membrane, which is a pivotal site (Magtanong et al., 2022). An increasing body of evidence suggests that ferroptosis constitutes a critical tumor suppressor mechanism.

## 3 Mechanism of ferroptosis

### 3.1 Depletion of glutathione (GSH)

Glutathione (GSH) is a tripeptide and a crucial cellular antioxidant. Glutathione peroxidase 4 (GPX4) is a glutathione-dependent antioxidant enzyme crucial in cell membrane repair, inflammation suppression, and facilitation of the iron death process. It stands as a pivotal regulatory factor in the process of ferroptosis (Li et al., 2019). GPX4 possesses a unique ability to reduce complex lipid hydroperoxides, simultaneously converting them to lipid alcohols, regardless of whether they are incorporated into biomembranes or lipoproteins (Brigelius-Flohe and Maiorino, 2013). Therefore, GPX4 is considered the sole glutathione peroxidase that safeguards biological membranes from oxidative damage and exerts a certain preventive effect on cellular ferroptosis (Brigelius-Flohe and Maiorino, 2013; Hassannia et al., 2019). Depletion of GSH can lead to GPX4 inactivation, subsequently causing cell membrane structural damage and cell demise (Zhang et al., 2023). Additionally, GSH depletion stands as a prominent feature of ferroptosis. As the transporter protein for glutamate/cysteine exchange (Xc) is essential for GSH synthesis, inhibiting Xc synthesis induces the occurrence of ferroptosis (Bertrand, 2017).

### 3.2 Influx of iron ions

Iron is an oxidation-active metal, and iron overload can damage organisms through a variety of mechanisms, including induction of ferroptosis in cells. Iron deficiency can increase the expression of cell cycle regulatory factors such as P15 and P20, thereby halting the division and growth of tumor cells in the G0/G1 phase (Rishi et al., 2021). Excess free ferrous ions within cells can react with oxygen and excessive free radicals, leading to the generation of radicals, lipid peroxidation, oxidative stress, and DNA damage (Cosialls et al., 2021). Therefore, an increase in intracellular iron content heightens the susceptibility of cells to ferroptosis (Hassannia et al., 2019). Additionally, studies have indicated that exogenous iron sources such as ferric ammonium citrate, ferric citrate, hexahydrated ferric chloride, among others, can also heighten the sensitivity of cells to ferroptosis (Dixon et al., 2012). A series of prior studies have shown that there are two independent coexisting mechanisms for promoting iron ion influx. One is the transferrin receptor 1 (TFR1)-dependent pathway reliant on transferrin, and the other is the hyaluronic acid (Hyal)-regulated pathway dependent on CD44, with the latter being more widespread and prevalent (Muller et al., 2020; Plays et al., 2021). Further investigations have discovered that in tumor patients, those with higher CD44 levels also exhibit relatively higher ferritin content (Rishi et al., 2021). Additionally, when the body’s iron levels rise, unlike the TFR1 gene, the expression level of the CD44 gene can increase in a positive feedback manner (Muller et al., 2020).

### 3.3 Regulation of lipid metabolism

The cellular lipidome consists of a diverse array of lipid species. Serving as primary substrates for peroxidation reactions, the cellular lipidome controls the abundance and availability of polyunsaturated fatty acids (PUFAs) to execute ferroptosis or modulate the sensitivity of cells to iron-induced cell death (Naowarojna et al., 2023). In the process of ferroptosis, the peroxidation of lipids extensively relies on the accumulation of endogenous iron within the body. From a biochemical perspective, the excessive accumulation of lipid peroxides in ferroptotic cells can inflict damage upon proteins, nucleic acids, and normal lipid macromolecules (Dixon et al., 2012; Cosialls et al., 2021). Research indicates that monounsaturated fatty acids (MUFAs) are less susceptible to peroxidation reactions due to the absence of pentadienyl groups and bis-allylic hydrogen atoms (Naowarojna et al., 2023). Consequently, the ratio of MUFAs to PUFAs significantly influences the sensitivity of cells to iron-induced cell death; when the MUFAs-to-PUFAs ratio is higher, the sensitivity to iron-induced cell death is diminished (Yang et al., 2016; Magtanong et al., 2022). Compared to free PUFAs molecules, enzymatically generated PUFAs-lipid complexes within the phospholipid bilayer of membranes are more prone to facilitating the diffusion of peroxide radicals and ferroptosis. In certain scenarios, the peroxidation of free PUFAs can also induce cellular ferroptosis (Zou et al., 2019; Naowarojna et al., 2023). Currently, research has confirmed that phospholipids and cholesterol play primary roles in mediating lipid peroxidation reactions and ferroptosis (Lee et al., 2021; Mortensen et al., 2023).

### 3.4 Imbalanced reactive oxygen species (ROS)

Most of the intracellular reactive oxygen species (ROS) originate from the superoxide radical. ROS initiate the oxidation of PUFAs. Among different types of ROS, the hydroxyl radical (OH•) possesses the highest chemical reactivity. It is a highly mobile, water-soluble ROS (Hassannia et al., 2019). Accumulated ROS are transformed into hydrogen peroxide (H2O2) by the action of superoxide dismutase (SOD). Upon reacting with ferrous ions, H2O2 undergoes the Fenton reaction, leading to the generation of hydroxyl radicals within various peroxidation groups (Wen et al., 2013). Simultaneously, intracellular ferrous ions convert the oxidative respiration products of the mitochondrial respiratory chain into toxic hydroxyl radicals, causing lipid peroxidation to reach lethal levels, damaging membrane structures, and inducing cell death (Xia et al., 2022). In normal cellular conditions, a balance is maintained between the generation and elimination of ROS, ensuring the proper metabolic activities of the organism’s cells. Excessive ROS, however, induce lipid peroxidation through a series of enzymatic reactions, ultimately leading to cell iron death (Su et al., 2019).

### 3.5 Calcium ion regulation

Calcium ions are so small in diameter that they can enter the cell with the help of transient changes in plasma membrane permeability. Therefore, the increase in intracellular calcium ion concentration can be considered an early indicator of membrane permeability alteration, with cell death being the most common outcome (Zhivotovsky and Orrenius, 2011). Research suggests that the activation of calcium channels is mediated by osmotic forces and is related to the formation of nanopores in the cell plasma membrane (Pedrera et al., 2021). The imbalance in intracellular and extracellular osmolarity, along with the activation of calcium channel signaling, is a downstream process in the molecular mechanism of cell iron death (Pedrera et al., 2023). Newer studies indicate that similar to the signaling pathways of iron death, calcium channel signaling can also propagate in a wave-like manner within a population of cells. This mode of propagation is independent of the osmotic differences across the cell membrane on both sides (Riegman et al., 2020). The storage of intracellular calcium, especially in the endoplasmic reticulum and mitochondria, plays a crucial role in the elevation of cytosolic calcium during cell iron death (Pedrera et al., 2023). The process by which extracellular calcium ion influx activates the release of intracellular calcium ions and lipid peroxidation, remains to be observed.

### 3.6 Regulation by P53

As a renowned tumor-suppressor gene, p53 regulates the ferroptosis process through its transcriptional activity or transcription-independent mechanisms, aiming to maintain cellular integrity (Kang et al., 2019; Liu et al., 2020b). Research has shown that P53 can inhibit the expression of the Xc system or promote spermidine/spermine N1-acetyltransferase 1 (SAT1), further activating arachidonate 15-lipoxygenase (ALOX15) expression (Chen et al., 2021a). Additionally, P53 sequesters dipeptidyl peptidase 4 (DPP4) within the cell nucleus to inhibit DPP4-dependent lipid peroxidation reactions, thus suppressing ferroptosis (Xie et al., 2017). Moreover, studies have confirmed that P53 can regulate iron death in ferroptosis-resistant tumor cells through autophagy processes (Cosialls et al., 2021). Additionally, research by Fujihara and colleagues revealed that mutant p53 activators like APR-246 and PRIMA-1MET induce non-dependent p53 ferroptosis by depleting GSH(Fujihara et al., 2021). Currently, a substantial body of research has identified P53 as a marker gene for ferroptosis.

Apart from the various regulatory factors mentioned above, certain biological processes are also involved in modulating cellular ferroptosis. Under normal circumstances, cells can protect themselves from various damages such as starvation, hypoxia, and drug toxicity through the process of autophagy. Unrestricted autophagy, however, can lead to cell death. Research by Liu et al. has revealed that the autophagy inducer rapamycin can deactivate mammalian target of rapamycin (MTOR) and degrade GPX4. This suggests that autophagy can regulate cellular ferroptosis by degrading GPX4 (Xie et al., 2021). Increasing evidence suggests that ferroptosis is a type of autophagy-dependent cell death, and elevated autophagic activity selectively promotes ferroptosis by degrading antioxidant proteins or organelles (Zhou et al., 2020). Firstly, activators of ferroptosis can lead to the formation of autophagosomes and an increase in autophagic flux. Secondly, the increase of autophagosomes in cells is positively correlated with ferroptosis sensitivity. Finally, the absence of core autophagy regulatory genes such as ATG5, ATG7, and BECN1 inhibits the process of ferroptosis (Gao et al., 2016; Liu et al., 2020a; Chen et al., 2023a). Upstream autophagy regulatory factors like TMEM164, downstream autophagy receptors such as HPCAL1, or danger signals like DCN all participate in mediating cellular ferroptosis within the autophagy-regulating pathway (Chen et al., 2023a).

## 4 The regulatory mechanisms of ferroptosis in GC cells

### 4.1 Ferroptosis-related regulatory pathways

#### 4.1.1 Xc/GSH regulatory pathway

GPX4 depends on GSH. It prevents cellular ferroptosis by reducing toxic lipid hydroperoxides to non-toxic lipid alcohols (Zhang et al., 2022). As previously mentioned, Xc is an essential protein for GSH synthesis, and the synthesis of GPX4 depends on GSH. Thus, inhibiting Xc synthesis can induce cell ferroptosis through the Xc/GSH pathway (Bertrand, 2017).

#### 4.1.2 Ferroptosis inhibitor protein 1 (FSP1) regulatory pathway

**FIGURE 1:**
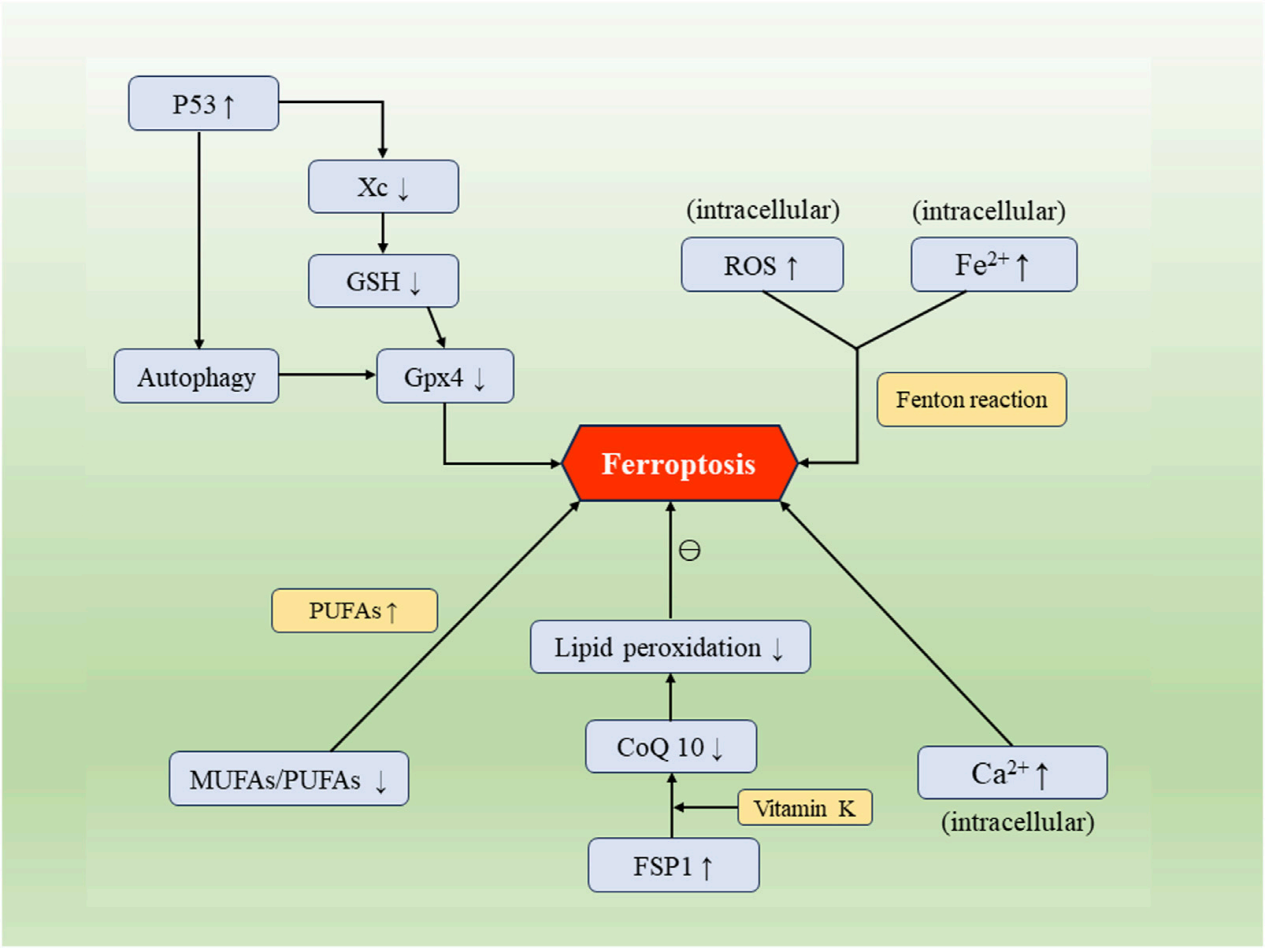
The occurrence and regulatory mechanisms of cellular ferroptosis. Factors such as Xc/GSH, FSP1, p53, along with biological processes including intracellular iron influx, calcium influx, disruption of lipid metabolism, and autophagy, can mutually regulate and impact the process of cellular ferroptosis.

FSP1 is one of the main regulatory molecules in ferroptosis. It inhibits lipid peroxidation through an alternative pathway distinct from glutathione dependency (Li et al., 2023c). FSP1 engages in redox reactions with vitamin K through the FSP1-CoQ10-NAD(P)H axis, leading to a reduction in CoQ10 content and subsequently reducing cellular lipid peroxidation rates to prevent the occurrence of cell ferroptosis (Bersuker et al., 2019) (Figure 1).

#### 4.1.3 Dihydroorotate dehydrogenase (DHODH) regulatory pathway

DHODH is localized on the outer surface of the mitochondrial membrane and serves as an inhibitor of ferroptosis. It can suppress ferroptosis in tumor cells, and cancer cell lines with DHODH gene deletion exhibit increased sensitivity to inducers of ferroptosis (Li et al., 2023a). DHODH functions by reducing Coenzyme Q (CoQ) to CoQH2 within the mitochondria, thereby inhibiting ferroptosis. Inactivation of DHODH can induce mitochondrial lipid peroxidation with reduced expression of GPX4 in tumor cells, leading to the occurrence of ferroptosis (Mao et al., 2021a; Mishima et al., 2023).

#### 4.1.4 Voltage-dependent anion channel (VDAC)

**FIGURE 2:**
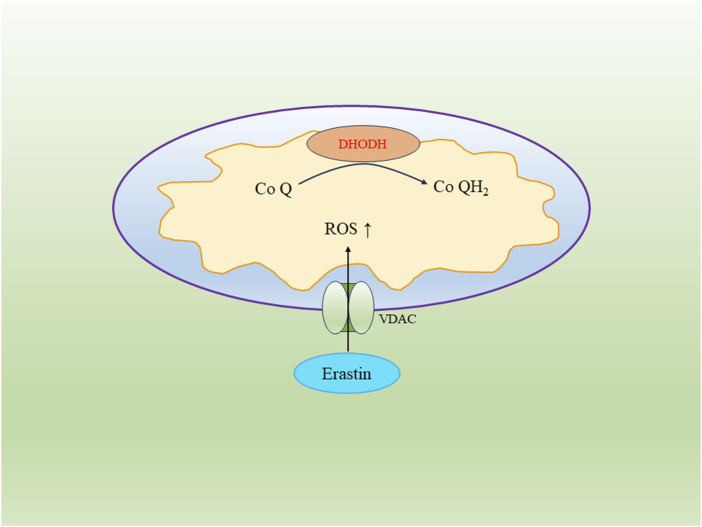
The regulatory pathway of DHODH and VDAC in the mitochondrion.

VDAC, known as the voltage-dependent anion channel, belongs to the eukaryotic mitochondrial porin protein family. It constitutes a significant portion of the outer mitochondrial membrane and regulates the exchange of substances between the mitochondria and the cytoplasm (He et al., 2022). In cancer cells, elevated levels of free tubulin close VDAC to decrease the mitochondrial membrane potential. However, the ferroptosis inducer Erastin can reverse this process. Erastin’s action on VDAC opens up the channel, leading to an increase in mitochondrial membrane potential and the abundant generation of mitochondrial ROS, ultimately triggering cell ferroptosis (DeHart et al., 2018) (Figure 2).

### 4.2 Non-coding RNAs associated with ferroptosis in GC cells

#### 4.2.1 MicroRNA

MicroRNAs (miRNAs) are a class of single-stranded RNA molecules consisting of 22–24 nucleotides. Several studies have demonstrated that miRNAs can trigger ferroptosis in GC cells. Experiments conducted by Ni et al. demonstrate that miR-375 can induce ferroptosis in GC cells both *in vivo* and *in vitro* by targeting SLC7A11. The miR-375/SLC7A11 regulatory axis serves as a potential target, capable of triggering ferroptosis and reducing the stemness of GC cells (Ni et al., 2021). Furthermore, Mao et al. found that SLC7A11, a transmembrane protein, is also a target of miR-489-3p. The miR-489-3p/SLC7A11 axis represents another pathway that can induce ferroptosis in GC cells (Mao et al., 2021b). Other research has revealed that the loss or downregulation of miR-221-3p can upregulate downstream activating transcription factor 3 (ATF3), leading to ferroptosis in GC cells. Upregulated ATF3 can inhibit the transcription of GPX4 and HRD1, further suppressing the proliferation of GC cells. HRD1 itself can inhibit ferroptosis by mediating the ubiquitination and degradation of ACSL4 (Shao et al., 2023). Moreover, miR-103a-3p has been found to be highly expressed in GC cells and promotes their proliferation. Glutaminase 2 (GLS2), targeted by miR-103a-3p, can be downregulated to inhibit GC cell proliferation and promote ferroptosis (Hu et al., 2018; Wu et al., 2020).

**FIGURE 3:**
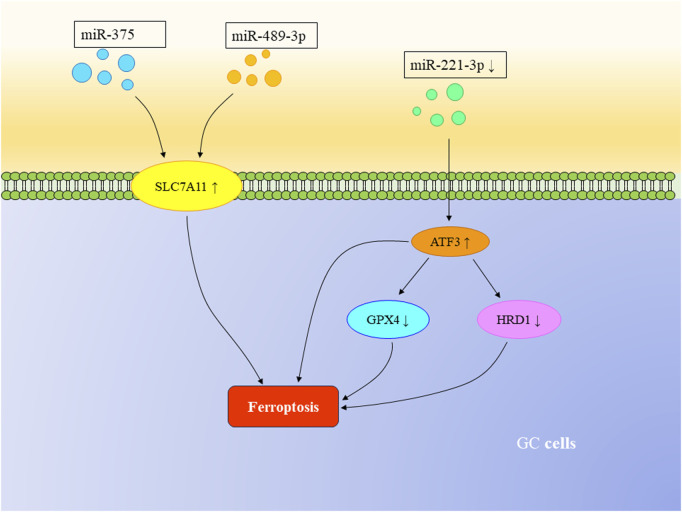
The regulatory role of mRNA in ferroptosis in GC. Both miR-375 and miR-489-3p can target SLC7A11, triggering ferroptosis in GC cells. The loss or downregulation of miR-221-3p can upregulate downstream ATF3, suppressing the transcription of GPX4 and HRD1, thereby inducing ferroptosis in GC cells.

Furthermore, in the previously mentioned miR-221-3p/ATF3 pathway, ATF3 has been identified as a downstream mRNA target of miR-221-3p (Shao et al., 2023). Relevant studies have confirmed that miRNAs can regulate mRNA through binding to the 3′untranslated region and can interact with other non-coding RNAs to mutually regulate ferroptosis in GC cells (Mao et al., 2017) (Figure 3).

#### 4.2.2 LncRNA

Long non-coding RNAs (lncRNAs) are a special type of RNA, characterized by their length exceeding 200 nucleotides, and they do not encode proteins. Despite this, lncRNAs are considered functional molecules that regulate gene expression at various levels, including chromatin, transcription, and post-transcriptional levels (Jiang et al., 2021). Pelaton is a type of lncRNA. Studies have shown that it acts as an inhibitor of ferroptosis. It exerts its role in inhibiting ferroptosis by suppressing P53 expression and mediating the ROS-dependent iron death pathway (Fu et al., 2022). Zhang et al.’s research revealed that an exosomal lncRNA called lncFERO derived from GC cells can control the tumorigenicity of GC stem cells by inhibiting cellular iron death through stearoyl-coenzyme A desaturase 1 (SCD1). This suggests that targeting the exosomal lncFERO/hnRNP A1/SCD1 axis in combination with chemotherapy could be a promising therapeutic approach based on GC stem cells (Zhang et al., 2021b).

Zheng et al. identified five lncRNAs associated with ferroptosis that can accurately predict the prognosis of GC patients and regulate the proliferation of GC cells. The results of signal detection for these five lncRNAs showed that high expression of RP11-1143G9.5 is associated with a favorable prognosis, while high expression of LINC00460, miR205HG, AC103563, and RP11-186F10.2 is associated with an unfavorable prognosis (Zheng et al., 2023). Wang et al. developed an lncRNA model related to ferroptosis that predicts the prognosis of patients with gastric adenocarcinoma. This model can predict Overall Survival (OS) based on 12 key lncRNAs and Progression-Free Survival (PFS) based on 13 key lncRNAs (Wang et al., 2022a). These ferroptosis-related lncRNA prediction models can serve as prognostic indicators for GC patients, offering potential avenues for precision treatment in the future.

#### 4.2.3 CircRNA

Various circular RNAs (circRNAs) are a type of covalently closed continuous circular non-coding RNA. circRNAs have been proven to play a significant regulatory role in the occurrence and progression of GC. Here, we have summarized the mechanistic roles of circRNAs associated with ferroptosis in GC. As previously mentioned, SLC7A11 is a direct target of miR-375 that induces ferroptosis in GC cells. Recent research has revealed that in GC cells, miR-375 can directly interact with circRPPH1. The latter is highly expressed in GC cells and relies on the miR-375/SLC7A11 axis to enhance the stemness of GC cells (Liu et al., 2023). Gao et al. found that circ0008035 acts as a sponge for miR-302a, thereby increasing the expression of downstream protein target E2F7 in GC cells. This inhibition suppresses the occurrence of ferroptosis in GC cells (Gao and Wang, 2023).

### 4.3 Tumor microenvironment in GC

**FIGURE 4:**
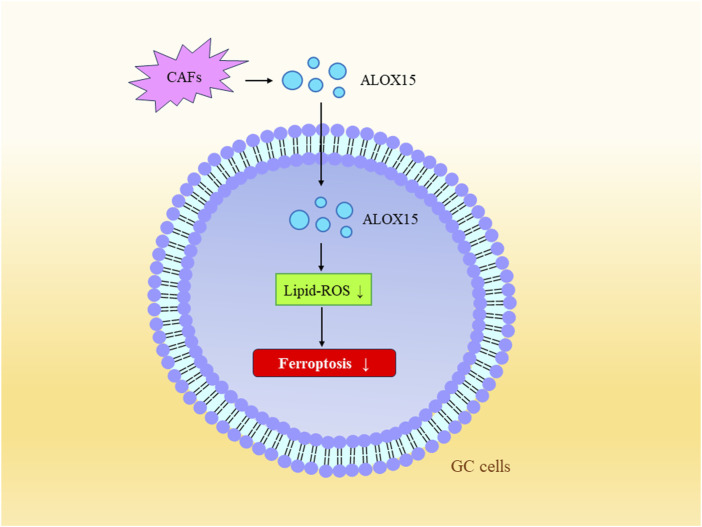
Tumor Microenvironment and Ferroptosis. Exosomal miR-522 secreted by CAFs inhibits the expression of ALOX15, consequently suppressing lipid-ROS production in gastric cancer cells, further preventing ferroptosis in these cells.

The tumor microenvironment (TME) is characterized by low oxygen levels, acidity, inflammation, and immune suppression. It involves various components such as cancer-associated fibroblasts (CAFs), vascular endothelial cells, and a certain number of immune cells (Xia and Quan, 2023). These stromal cells release various signaling molecules that can activate tumor cell proliferation or remodel the surrounding area (Mao et al., 2017). Studies have also found that tumor cells and their surrounding microenvironment can establish different rates of ferroptosis activation (Chen et al., 2021b). Under hypoxic conditions, hypoxia-inducible factor 1-alpha (HIF-1α)/lncRNA-PMAN prevents ferroptosis in GC cells by enhancing the stability of SLC7A11 mRNA (Lin et al., 2022). Hypoxia-induced lncRNA CBSLR and the CBS signaling axis reduce the methylation of ACSL4 protein through polyubiquitination, leading to the degradation of ACSL4 protein. This protects GC cells from ferroptosis and contributes to chemoresistance (Yang et al., 2022; Xia and Quan, 2023). CAFs are a critical component of the tumor microenvironment and serve as sources of cytokines, growth factors, and exosomes (Sahai et al., 2020). Research has found that within the tumor microenvironment of GC, CAFs suppress the expression of ALOX15 in cancer cells by secreting exosomal miR-522. This action inhibits lipid-ROS production in cancer cells, thereby preventing ferroptosis in GC cells (Zhang et al., 2020) (Figure 4).

Early studies found that tumors with an immune-excluded phenotype contain a large number of immune cells in their surrounding stroma, but these immune cells do not effectively carry out immune functions (Chen and Mellman, 2017). However, current research suggests that the formation of an immune-inflammatory tumor microenvironment primarily originates from genomic alterations in the tumor, such as microsatellite instability (MSI) and tumor mutation burden (TMB). These alterations not only lead to the generation of more neoantigens by tumor cells, attracting a substantial infiltration of tumor-infiltrating lymphocytes (TILs) into the tumor microenvironment to attack tumor cells, but also reveal a strong negative correlation between the extent of ferroptosis in GC cells and TMB(Kandoth et al., 2013; Charoentong et al., 2017; Zhang et al., 2021a). ALB, which encodes albumin, is widely regarded as a prognostic indicator for GC. ALB and TP53 are suggested to be potential links connecting GC cell ferroptosis and TMB(Zhang et al., 2021a).

## 5 Ferroptosis and treatment of GC

5-Fluorouracil (5-FU) is a commonly used chemotherapy drug that kills tumors by interfering with DNA and RNA synthesis. However, due to factors such as tumor recurrence, metastasis, and angiogenesis, the number of patients resistant to 5-FU is increasing (Sethy and Kundu, 2021). Baicalin is an effective component of the traditional Chinese medicine Scutellaria baicalensis. Research by Yuan et al. revealed that baicalin promotes the generation of ROS in GC cells, inducing ferroptosis in these cells, and it can also inhibit resistance to 5-FU, enhancing its anti-tumor effects (Yuan et al., 2023). Therefore, the combination of baicalin and 5-FU offers a new approach for the treatment of advanced GC. A relevant review article suggests that targeting the steady-state of ROS may provide an effective therapeutic strategy for targeting drug-resistant GC cells, as prolonged high ROS levels can lead to resistance (Gu et al., 2022). Hu et al. discovered that Platycodin B (PB) is a novel GPX4 inhibitor that induces ferroptosis in GC cells by downregulating GPX4 expression. PB demonstrated effective anti-tumor properties in both GC cells and mouse models without significant toxicity to the host (Hu et al., 2023). Therefore, PB could be a promising potential therapeutic agent for GC treatment. Arenobufagin (ArBu) is a bufadienolide isolated from the skin and venom of toads, known for its broad anti-tumor properties. Research has shown that ArBu induces ferroptosis in GC cells by targeting and enhancing the expression of Rev-erbα(Chen et al., 2023b). Moreover, ArBu has the potential to enhance sensitivity to cisplatin chemotherapy, making it a promising candidate for preventing GC progression and alleviating chemoresistance (Si et al., 2022). Similarly, various studies have indicated that Ophiopogonin B (OP-B), capecitabine, apatinib, and artemisinin can induce ferroptosis in GC cells, inhibit cell proliferation, and exhibit therapeutic effects (Zhao et al., 2021; Zhu et al., 2021; Geng and Wu, 2022; Zhang et al., 2022).

Dexmedetomidine (DEX) is a commonly used anesthetic agent that has been found to inhibit GC cell activity *in vitro* and suppress cancer cell growth *in vivo*. Further research has shown that DEX increases intracellular ROS and iron content while reducing the levels of GSH and GPX4 in GC cells. DEX can induce ferroptosis in GC cells by regulating the circ0008035/miR-302a/E2F7 axis (Gao and Wang, 2023). Another local anesthetic, levobupivacaine, also possesses potential anti-tumor properties. As previously mentioned, the miR-489-3p/SLC7A11 axis can regulate ferroptosis in GC cells. Levobupivacaine can upregulate miR-489-3p, which induces ferroptosis by targeting SLC7A11, thereby inhibiting GC cell proliferation (Mao et al., 2021b). Studies have revealed that ubiquitin-specific protease 7 (USP7) stabilizes heterogeneous nuclear ribonucleoprotein A1 (hnRNPA1) through deubiquitination, mediating the secretion of miR-522 in CAFs. First-line chemotherapeutic agents such as cisplatin and paclitaxel activate the USP7/hnRNPA1 axis, leading to miR-522 secretion, which downregulates ALOX15 expression in GC cells, inhibiting ferroptosis and ultimately reducing chemotherapy sensitivity (Zhang et al., 2020). Hence, inhibition of miR-522 secretion has the potential to restrain tumor proliferation and heighten susceptibility to cisplatin and paclitaxel, presenting a viable therapeutic strategy. Propofol similarly inhibits the malignant phenotype of GC cells by regulating the miR-125b/STAT3 axis, inducing ferroptosis in GC cells (Liu et al., 2021). Likewise, Mao et al. confirmed that levobupivacaine can induce ferroptosis in GC cells via the miR-489-3p/SLC7A11 signaling pathway (Mao et al., 2021b). These findings hold potential value in the diagnosis and treatment of GC.

In the realm of traditional Chinese medicine, some herbal remedies also exhibit similar effects. Song et al. demonstrated that the Yiqi Huayu Decoction induces ferroptosis in GC cells through the JAK2-STAT3 pathway and ACSL4 expression, which helps prevent metastasis or recurrence of GC (Song et al., 2022). Tanshinone IIA is an active ingredient extracted from Danshen, and it can induce ferroptosis in GC cells by upregulating the P53 pathway, thereby inhibiting the stemness of GC cells (Ni et al., 2022). Chu et al. confirmed that Fuzheng Ningzeng Decoction (FZNZ) can be used to treat gastric precancerous lesions. FZNZ induces lipid peroxidation and mitochondrial damage in precancerous cells, increases cellular labile iron and ROS levels, and decreases cellular GPX4/GSH levels. Additionally, FZNZ can induce endoplasmic reticulum stress, which also participates in regulating cellular ferroptosis (Chu et al., 2022). Based on their research on key gene targets TLR4 and KRAS, Li et al. affirmed that Resveratrol and Magnolol offer dual therapeutic and preventive benefits for GC patients. Furthermore, other traditional herbal medicines like Curcumin, Salvianolic acid B, Coptis chinensis, and Rhizoma polygoni cuspidati provide avenues and alternative approaches for immune modulation of the TME and ferroptosis in GC cells (Li et al., 2023b).

## 6 Summary and prospect

The primary treatment for GC remains surgery; however, postoperative recurrence is also more common. Diagnosis and treatment of GC have consistently been among the key challenges in the medical field. Ferroptosis, as a novel form of cell death, is currently a hot research topic. Overall, the occurrence of ferroptosis in GC cells significantly impacts their proliferation, invasion, metastasis, and other behaviors. Based on existing research findings, we have discussed the mechanisms of action of a series of anti-tumor drugs. In recent years, research has provided new insights into the molecular mechanisms of ferroptosis. As we have previously explained, some ferroptosis-related genes can serve as biomarkers for predicting GC. However, the existence of ferroptosis biomarkers that can determine the severity of GC still requires further exploration. Pathways of ferroptosis mediated by specific biological molecules could potentially serve as new targets for GC treatment and prognosis, offering valuable insights for precision therapies in the future.
